# Bacterial translocation to mesenteric lymph nodes fueling surgical site infections: evidence, technical challenges and future directions

**DOI:** 10.1186/s12967-025-06462-x

**Published:** 2025-08-05

**Authors:** Simone N. Zwicky, Lara Mordasini, Daniel Spari, Bahtiyar Yilmaz, Guido Beldi

**Affiliations:** Department of Visceral Surgery and Medicine, Inselspital, Bern University Hospital, University of Bern, Freiburgstrasse 18, 3010 Bern, Switzerland

**Keywords:** Surgical site infections, Bacterial translocation, Abdominal surgery, Metagenomic sequencing, Whole genome sequencing, PCR

## Abstract

Surgical site infections (SSIs) continue to pose a significant healthcare challenge by contributing to longer post-surgical recovery times, greater healthcare costs and higher patient mortality. The traditional understanding of SSIs has focused on the impact of various external origins of contamination or on the importance of intestinal spillage during surgical procedures. However, recent studies highlight the significant contribution of the patient's intestinal microbiota in the onset of SSIs. One possible pathway of infection is translocation of bacteria from the intestines to organs that are typically sterile, such as the mesenteric lymph nodes (MLNs). These secondary lymphoid organs are then potential reservoirs for SSIs. This review summarizes the current data on the incidence and mechanisms of bacterial translocation (BT) to MLNs in the context of a surgical insult and its association with postoperative infectious complications. Data from animal studies discuss how BT to MLNs is driven by factors such as dysbiosis and surgical interventions and is strongly linked to infectious outcomes. Potential translocation pathways including intracellular transit and carrier-independent mechanisms are explored. Similarly, human studies provide evidence that BT to MLNs is a frequent occurrence during abdominal surgery and significantly increases the risk of infectious complications. We further discuss the limitations of current methodologies for studying BT and SSIs and highlight how advanced techniques can provide novel insights into these processes. This review identifies key areas for future research and potential targets for preventative strategies to increase our understanding of the role of the intestinal microbiota in BT to MLNs and its contribution to SSIs.

## Introduction

### Surgical site infections remain common after abdominal surgery

Approximately 313 million surgical procedures are performed worldwide each year [1]. Despite medical advancements, postoperative mortality remains a significant concern ranking as the third leading cause of deaths worldwide [2]. Each year, approximately 4.2 million individuals die within 30 days of surgery [2], accounting for 7.7% of all global fatalities [3]. Among these fatalities, surgical site infections (SSIs) ranging from superficial to organ/space play a predominant role [4, 5]. In highly industrialized countries, up to 10% of patients undergoing gastrointestinal surgery develop SSIs within 30 days postoperatively [6]. Beyond their impact on mortality, SSIs impose a substantial economic burden with an estimated cost of 3 to 10 billion U.S. dollars to the U.S. healthcare system annually [7, 8]. Further complicating efforts to prevent SSI, 16–35% of SSIs involve pathogens resistant to the prophylactic antibiotics administered during surgery [6].

Efforts to prevent SSIs have led to the development of comprehensive surveillance programs and evidence-based preventive measures [9]. Key strategies include the timely administration of perioperative antibiotic prophylaxis, the use of appropriate antiseptic solutions, and the avoidance of unnecessary measures such as hair removal, which can increase the risk of infection [9]. Despite these preventive measures, the incidence of SSIs remains unacceptably high, particularly in high-risk surgical populations. Infection rates vary significantly by the type of surgery, patient comorbidities, and the infrastructure of the healthcare system [6, 10].

### Bacterial origins of surgical site infections and the unknown routes from the patient’s microbiota

**Fig. 1:**
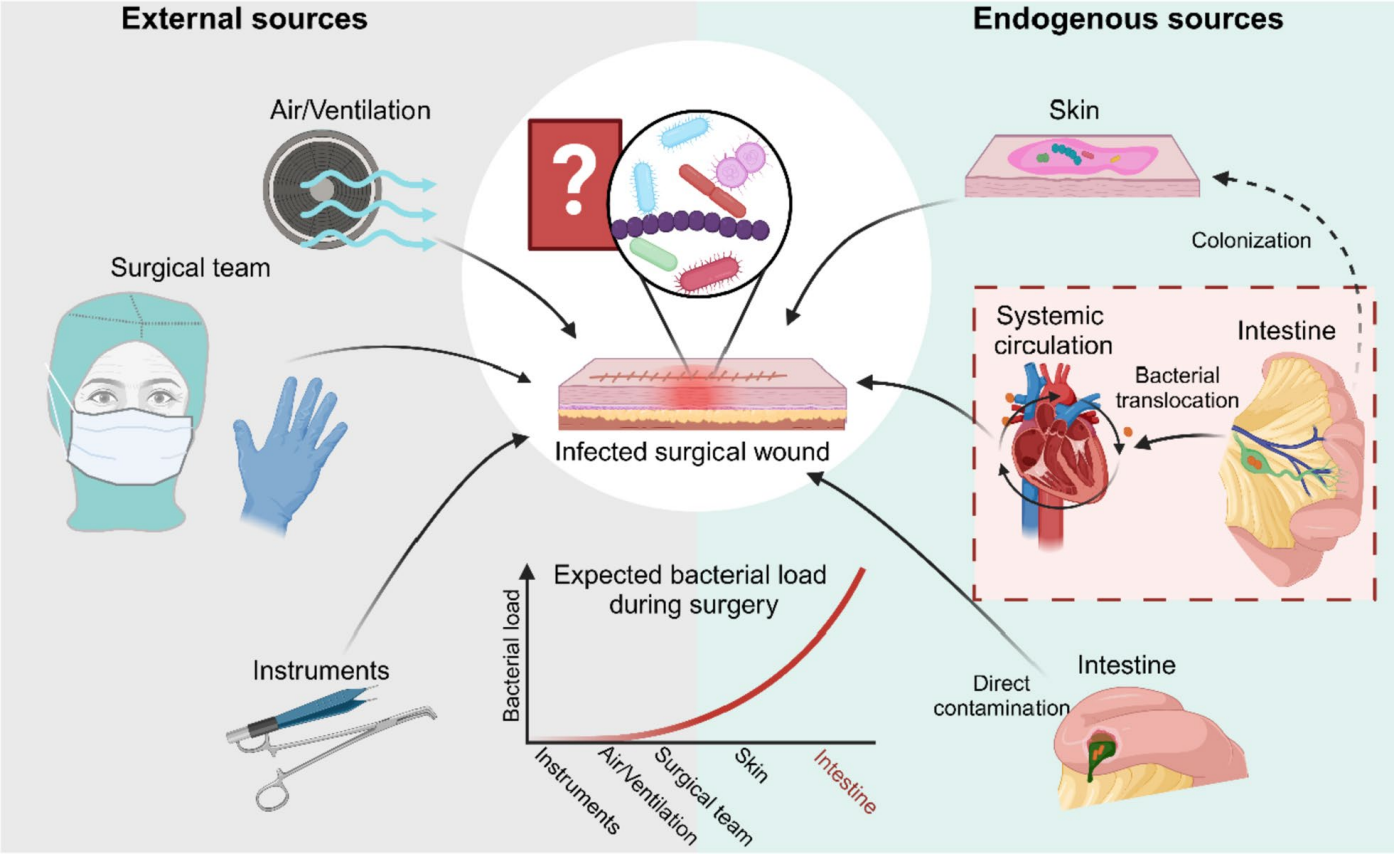
Potential origin of SSI-pathogens. The origin of pathogens responsible for SSIs remains a subject of debate. Potential external  sources include airborne contamination from the air or ventilation system, microbial transfer from the surgical team, and instruments. Endogenous sources primarily include the patient’s skin and intestinal microbiota. Pathogens from the intestine may reach the surgical site through translocation into the systemic circulation or via direct contamination in cases of intraoperative intestinal spillage. At the time of surgery, the highest bacterial load is found in the patient’s intestinal tract, followed by the skin, which undergoes disinfection prior to the procedure. Among external sources, the surgical team carries a higher bacterial load compared to the air in the operating room, and surgical instruments. Emerging research from environments in which external sources are highly controlled, suggests that bacteria causing SSIs originate from the patient’s own microbiota, though the exact source remains unproven. Created in BioRender. Zwicky, S. (2025) https://BioRender.com/mhz3xvw

The pathogenesis of SSIs is complex, and the source and route of infection are debated. Traditionally, wound infections are attributed to contamination during surgery, either from external sources in the operating room, or endogenous contamination from intraoperative events such as intestinal spillage [15] (Fig. 1). To minimize the risk of infection from external sources, sterilization of instruments [16] and room ventilation are essential [16, 17]. To reduce contamination by the surgical team aseptic techniques, hand hygiene, sterile gloves, masks, and gowns, were widely implemented [9]. Consequently, the bacterial load on external sources remains extremely low, making the endogenous source of infection far more probable given the high numbers of bacteria present on the patient.

Surgical procedures are classified by contamination risk into clean, clean-contaminated, contaminated, and dirty/infected categories, with contaminated and dirty/infected procedures having a higher risk of SSIs [18]. Interestingly, despite such associations, bacterial contamination of the wound, as indicated by a positive culture, seems not to reliably correlate with subsequent clinical infection [19–21].

Gastrointestinal surgeries are categorized as clean-contaminated, contaminated or even dirty/infected procedures and have SSI rates from 10% [6] to 30% [22]. In contrast, non-gastrointestinal surgeries, typically classified as clean, report lower SSI rates, from about 1% for knee surgery to 10% for complex cardiac surgery [23, 24].

The incidence of SSIs following gastrointestinal surgery has been stratified by the United Nations Human Development Index, HDI [6]. The reported SSI incidence is 9.4% in high-HDI countries, 14.0% in middle-HDI countries, and 23.2% in low-HDI countries [6]. After adjusting for intraoperative contamination events, SSI incidence remains significantly higher in lower-HDI countries. This data suggests that HDI itself is an independent risk factor for SSIs, highlighting the role of broader systemic factors beyond intraoperative events [6].

In conclusion, contamination from external sources or endogenous contamination due to breach in sterility or intraoperative exposure to colonized organs and cavities alone does not fully explain SSI risk [6, 15, 18–21]. Even with proper sterilization and the management of external sources of infection, SSIs remain a clinical problem, thus highlighting that other factors beyond contamination contribute to the risk of infection [6, 25, 26].

### The patient’s own bacteria as origin of infection

A recent study reporting on clean spinal surgeries and used advanced sequencing methods is one of the first to support the hypothesis that bacteria identified in SSIs may predominantly originate from the patient’s own microbiota [27]. In this study, samples were collected from the rectum, nose, and skin prior to surgery [27]. In the event of a SSI, the isolated pathogens from the wound culture were compared with the preoperative samples [27]. The researchers showed that most of the pathogens found in SSIs are genetically similar to the patient’s own bacteria present at the start of surgery. Although reliable strain-level comparisons were lacking (Bacterial enrichment to reduce host-associated noise), their data suggests the origin of SSI pathogens is from the patient's own microbiome [27]. The intestine and skin are the main suspects as the source of SSIs due to their high colonization rate. In the study discussed above, the reported SSI isolates matching preoperative microbiota were mainly intestinal bacteria, such as *Escherichia coli* (*E. coli*) and *Enterococcus *spp. along with *Staphylococcus aureus *(*S. aureus*) [27]*.* While the study postulates the skin as reservoir of wound infection, it’s important to note that preoperative skin swabs were collected before surgical skin disinfection, potentially capturing transient intestinal bacteria on the skin, which may not represent the true bacterial profile at the time point of the actual surgical incision [27].

The bacteria associated with SSIs also varies depending on the type of surgery. The most frequently reported pathogens across all surgeries are *S. aureus*, *E. coli*, and *Enterococcus *spp. [28]. While Staphylococci predominate in SSIs following extra-abdominal procedures such as orthopedic surgeries (about 40%), *E. coli* and *Enterococcus *spp. are more frequently associated with abdominal surgeries [28]. Remarkably, typical intestinal bacteria like *Enterococcus faecalis *(*E. faecalis*) are also found in 5–30% of orthopedic SSIs [10].

Notably, recent studies suggest that even *S. aureus*, a pathobiont traditionally linked to the skin, predominantly colonizes the intestine rather than the skin or nose [29, 30]. Given that the skin is disinfected prior to surgery, it is possible that *S. aureus* found in SSIs originates from the intestine.

In conclusion, there is growing evidence suggesting the patient’s own microbiota is the primary source for SSIs [27]. Bacteria colonizing the intestine are highly prevalent in SSIs across many surgery types, making an intestinal origin of SSIs likely. However, the precise origin within the patient's own microbiota has yet to be reliably demonstrated. The high number of intestinal bacteria in SSIs following “clean” surgeries without any intestinal manipulation or breach in sterility suggests alternative routes of infection [31]. One plausible mechanism is bacterial translocation (BT), in which bacteria originating from the patient's intestine migrate to distant sterile sites, such as the surgical incision and contribute to infections.

## Bacterial translocation as a route of infection

**Fig. 2:**
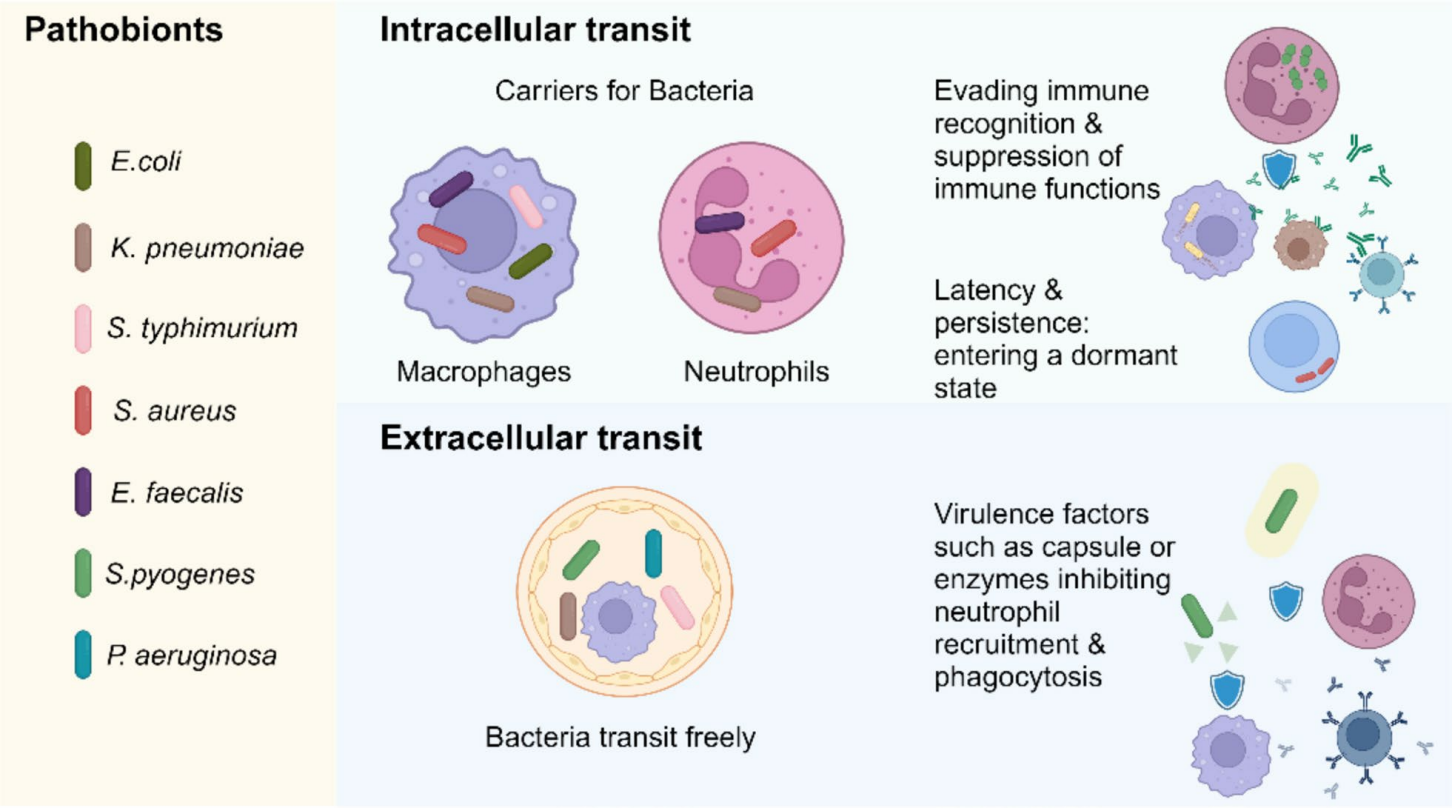
Different potential ways of dissemination of pathobionts from the intestine to SSIs. Studies reporting ways of dissemination for: *E. coli* [35–38], *K. pneumoniae* [39, 40], *S. typhimurium* [41, 42], *S. aureus* [43–46], *E. faecalis* [47–49], *S. pyogenes* and *P. aeruginosa* [50]. Transit in the systemic circulation occurs via intra- and extracellular transport. Intracellular transit within professional phagocytic cells allows pathobionts to evade immune recognition and suppress host defenses. During extracellular transit, pathobionts are protected by virulence factors, such as capsules or proteases enhancing their extracellular survival in hostile environments. Created in BioRender. Zwicky, S. (2025) https://BioRender.com/77tlio8

The potential for bacteria to cross the gastrointestinal epithelium was first described in electron microscopy studies in the 1960s [32]. *Berg et al.* introduced the concept of BT in 1979, which is that bacteria can migrate from the intestine to sterile tissues, such as the mesenteric lymph nodes (MLNs), spleen, and liver [33, 34]. The following sections will discuss ways of systemic dissemination underlying BT to MLNs, including intra- and extracellular transit (Fig. 2), and its contribution to infectious complications, primarily using evidence from animal studies.

### Duality in bacterial dissemination

Bacteria are characterized as either thriving outside cells (extracellular), requiring a host cell for survival (obligate intracellular), or invading host cells to gain a selective advantage (facultative intracellular) [51, 52] (Fig. 2). An increasing number of taxa, previously believed to be strictly extracellular, have demonstrated a facultative intracellular behavior [52]. These include pathobionts often involved in SSIs such as *E. faecalis* [47–49, 53], *E. coli* [35–37], *S. aureus* [43–45]. Reports indicate that *E. faecalis* can persist in non-professional phagocytic cells, including epithelial cells, as well as in professional phagocytic cells like neutrophils and macrophages [47–49]. A recent study has reported that *E. faecalis* not only persists but also actively replicates within parenchymal cells such as hepatocytes [53].

To survive in lymphatic tissue, whether intracellularly or extracellularly, bacteria require virulence factors to evade phagocytic killing by preventing neutrophil and macrophage recruitment or survive upon phagocytosis [54]. Intracellular bacteria subvert host immune recognition and modulate or suppress immune functions by many mechanisms [55]. For example, *S. aureus* upregulates the “don't eat me” signal CD47 [56], and *Klebsiella pneumoniae *(*K. pneumoniae*) decreases the “eat me” signal phosphatidylserine, PS [39], reducing their clearance by macrophages. When engulfed by macrophages, *K. pneumoniae* survives by phagosome manipulation [40]. Extracellular *Streptococcus pyogenes *(*S. pyogenes*) survives by employing virulence factors such as the hyaluronan capsule and the chemokine-cleaving protease “*S. pyogenes* Cell-Envelope Proteinase” (SpyCEP), which facilitate lymphatic dissemination and inhibit neutrophil recruitment, allowing them to evade the immune response and transit through lymphatic system [57].

In the following two sections, we discuss how intra- and extracellular dissemination of pathobionts, contributes to BT and the pathogenesis of SSIs.

### Hiding in cells? Intracellular transport as the route of infection

Drawing inspiration from Greek mythology the “Trojan Horse hypothesis” suggests that immune cells, such as neutrophils or macrophages, serve as unwitting carriers, stealthily transporting intestinal-derived bacteria through systemic circulation to surgical wounds [58].

Methicillin-resistant *S. aureus* (MRSA) has been detected within neutrophils in the bloodstream and subsequently in surgical wounds [45]. In a murine model, oral administration of MRSA combined with antibiotic-induced dysbiosis and surgical stress resulted in wound abscess formation. MRSA could be detected in the wound and contamination was ruled out through wound cultures [45]. Additionally, a rat model demonstrated that intestinal-derived MRSA is transported by neutrophils and leads to remote prosthetic infections [59].

CX3CR1+ mononuclear phagocytes can also take up and transport intestinal bacteria to MLNs under conditions such as antibiotic-induced dysbiosis [41]. Disrupting the intestinal microbiota with an antibiotic intervention increased translocation of both commensal and well-established facultative intracellular pathogenic bacteria, such as *Salmonella typhimurium *(*S. typhimurium*), to MLNs [41]. MLNs can act as a reservoir for *S. typhimurium*, leading to relapsing infections upon antibiotic withdrawal [60]. The resident intestinal microbiota protects against BT to MLNs as shown in germ-free and antibiotic-treated mice, in which the burden of *S. typhimurium* in the MLN was higher compared to conventionally raised mice [61].

Collectively, recent evidence and emerging insights lend support to the “Trojan Horse hypothesis”, showing that intracellular carriage is a plausible pathway for intestinal-derived microbiota to contribute to the development of SSIs.

### No need to hide? Extracellular translocation as the route of infection

Pathobionts disseminate not only through intracellular mechanisms but also via extracellular routes, traveling between body sites and contributing to infection. For instance, *S. typhimurium* has been shown to translocate from the intestine to MLNs either associated with dendritic cells or independently without a carrier [42]. Although evidence showing extracellular transport from the intestinal tract to MLNs is still limited [42], extracellular dissemination has been demonstrated in other contexts. For example, *S. pyogenes* traverses extracellularly from the site of infection through lymphatic vessels to regional and distant lymph nodes, subsequently entering the bloodstream without being engulfed by phagocytes [57]. Similarly, extracellular pathobionts like *K. pneumoniae* and *Pseudomonas aeruginosa* (*P. aeruginosa*) have been observed to travel to local and distant draining lymph nodes following intramuscular injection [50]. These findings suggest that the lymphatic system functions both as a conduit and a reservoir for occult bacteremia and distant soft tissue infections [57].

In summary, both intracellular and extracellular dissemination appear to represent critical pathways in the spread of infection (Fig. 2). However, the distinction between extracellular and intracellular modes of bacterial transport remains nuanced, as emerging evidence suggests that these mechanisms may coexist and bacteria interact with host cells in complex and interdependent ways [54].

## Bacterial translocation to mesenteric lymph nodes: evidence from human surgical studies

Beginning in the late 1980s, the first human studies began to report BT to MLNs during surgery [62, 63]. Since then, several studies, have investigated BT to MLNs in patients undergoing various abdominal surgeries, including simple laparotomies, colorectal cancer (CRC) surgery, pancreatoduodenectomies, and esophagectomies [62–79] (Table 1). Some of these studies compared BT to MLNs at the start and end of surgery [68, 70, 72–78]. At the start of surgery, BT to MLNs ranged from 0 to 56% (~ 20% on average) [63–65–69, 72–79]. At later timepoints of the surgery, after bowel mobilization, studies reported BT rates of around 19 to 80% (~50% on average) [68, 70–78]. Such a 2.5-fold increase in BT to MLNs may result from the mobilization and manipulation of the intestine. However, BT to MLNs has also been observed in surgeries without bowel mobilization such as major hepatectomies and abdominal aortic aneurysm repair [66, 70]. During hepatectomies there was an increase of bacterial DNA detected in MLNs when compared to start of the procedure (start 29% vs. end 37% MLNs positive for bacterial DNA) [70] and in patients undergoing abdominal aneurysm repair, in 10% of MLNs viable bacteria were identified [66]. Even in non-intestinal surgeries, typical intestinal bacteria such as *E. coli*, *Enterococcus* spp., *Klebsiella* spp., and *P. aeruginosa* were commonly identified in the MLNs [66]. No differences in BT rates were observed between open and laparoscopic surgery, suggesting that it is not the extent of manipulation, but rather the surgery and anesthesia itself that may lead to variances in BT [73, 77].

**Table 1:** Synopsis of human trials investigating BT to MLNs during gastrointestinal surgery

Population	Exclusion criteria	Sample sites	Bacterial identification technique	Prophylactic antibiotics, intestinal microbiota modulation	BT rate to MLN	Most often identified bacteria	Type of surgery	Clinical associated outcome with BT:^a^	Reference
Group A: CRC patients (n = 20) Group B: Non-CRC (n = 20)	None mentioned	MLN location: peri-colonic MLN timepoint: not specified; liver tissue, feces, portal blood	Culture, conventional microbial identification (counts, appearance)	IV antibiotics (not specified) post-sample collection	Group A: 13/20 (65%) Group B: 6/20 (30%)	Group A: *E. coli, Enterococcus *spp.,* P. aeruginosa, B. fragilis, Bifidobacterium *spp. Group B: *C. perfringens, E. coli, P. aeruginosa*	Colorectal resection	No follow-up for clinical outcomes	[62], **1988**
General surgery (n = 267)	Preoperative antibiotic treatment, unfeasible tissue sampling, rapid hemorrhage, adhesions in the terminal ileum	MLN location: terminal ileum MLN timepoint: pre-mobilization; serosal scraping, small bowel biopsy, venous blood	Culture, conventional microbial identification (counts, appearance), villous height analysis	Antibiotics not reported	21/242 (10.3%)	*E. coli, K. oxytoca, E. cloacae, B. fragilis, E.. faecalis, M. morganii*	Laparotomy	Higher infectious complications BT vs. Non-BT: 7/25 (28%) vs. 25/217 (11.5%), p < 0.05	[63], **1994**
General surgery (n = 448)	Preoperative antibiotic treatment (2 weeks), unfeasible tissue sampling, intraperitoneal sepsis/contamination	MLN location: terminal ileum MLN timepoint: pre-mobilization; serosal scraping, venous blood	Culture, conventional microbial identification (counts, appearance)	Antibiotics not reported	69/448 (15.4%)	*E. coli, E. faecalis, E. cloacae, CNS, B. fragilis, K. oxytoca*	Laparotomy	Higher infectious complications BT vs. Non-BT: 31/69 (45%) vs. 72/379 (19%), p < 0.001 Matching organisms in MLNs and septic foci (*E. coli, P. mirabilis, K. oxytoca, CNS*)	[64], **1998**
General surgery (n = 927)	Preoperative antibiotic treatment (2 weeks), unfeasible tissue sampling, intraperitoneal sepsis/contamination	MLN location: ileocolic MLN timepoint: pre-mobilization; nasogastric aspirates	Culture, conventional microbial identification (counts, appearance), bacterial identification strips	IV antibiotics (not specified) post-sample collection	130/927 (14%)	*Not mentioned*	Laparotomy	Higher infectious complications BT vs. Non-BT: 42.3% vs. 19.9%, p < 0.001 Higher wound infection rates in BT vs. Non-BT: 22/130 (16.9%) vs. 71/797 (8.9%), p-value not specified	[79], **2006**
General surgery (n = 279)	Preoperative antibiotic treatment, proton pump inhibitors, H2 receptor antagonists, preoperative enteral/parenteral nutrition (1 month), intraoperative contamination	MLN location: terminal ileum MLN timepoint: pre-mobilization; serosal scraping, nasogastric aspirates, venous blood	Culture, conventional microbial identification (counts, appearance), bacterial identification strips	IV antibiotics (not specified) post-sample collection	29/136 (21.3%)	*E. coli, E. faecalis, Enterobacter *spp., *Proteus *spp., *Bacillus *spp., *K. oxytoca, S. epidermidis* *Septic focus: E. coli, E. faecalis, Pseudomonas, S. epidermidis*	Laparotomy	Higher infectious complications BT vs Non-BT: 11/29 (38%) vs. 32/107 (30%), p = ns Proximal intestinal colonization associated with BT and septic morbidity Matching organisms in nasogastric aspirates, MLNs, and septic foci (*E. coli, Proteus *spp.,* E faecalis*)	[65], **1999**
Abdominal aortic aneurysm (n = 51)	Unfeasible tissue sampling	MLN location: ileocolic MLN timepoint: pre-mobilization; serosal exudate, mural thrombus	Culture, conventional microbial identification (counts, appearance)	IV antibiotics (Co-amoxiclav 1.2 g, Cefuroxime 1.5 g, Metronidazole 500 mg) post-sample collection	5/51 (10%)	*E. coli, E. faecalis, S. aureus, Klebsiella *spp.,* P. mirabilis, Streptococcus *spp. Septic focus: *E. coli, Klebsiella *spp.,* P. mirabilis, Streptococcus *spp.	Abdominal aortic aneurysm repair	Higher infectious complications BT vs. Non-BT: 4/5 (80%) vs. 9/46 (19.5%), p = 0.013 Matching organisms in 3/4 (75%) of MLNs and septic foci (*E. coli, P. mirabilis*)	[66], **2000**
Carcinoma cecal region (n = 10)	Signs of intestinal stenosis, pre-existing inflammatory disease, abnormal nutritional status/liver function/metabolism/cardiovascular performance	MLN location: ileocolic (2 per patient) MLN timepoint: pre-mobilization; venous blood	Culture, conventional microbial identification (counts, appearance), qPCR 16S gene *E. coli*, endotoxin assay	IV antibiotics (not specified) post-sample collection	Culture: 9/20 (45%) MLNs, 7/10 (70%) patients 16S rPCR: 6/20 (30%) MLNs, 4/10 (40%) patients	*E. coli, E. faecalis, CNS, S. aureus, Bacteroides *spp.,* Corynebacteria, S. mitis*	Right hemicolectomy	No Follow-up for clinical outcomes	[67], **2000**
Group A: historical controls elective surgery (n = 472) Group B: elective surgery (n = 50)	Preoperative antibiotic treatment (2 weeks), unfeasible tissue sampling, intraperitoneal sepsis/contamination	MLN location: Group A ileocolic, Group B: ileocolic/inferior mesenteric pedicle MLN timepoint 1: pre-mobilization, 2: post-mobilization	Culture, conventional microbial identification (counts, appearance), bacterial identification strips	IV antibiotics (Cefuroxime + Metronidazole) after induction of anaesthesia before surgery	Group A: 54/472 (11.4%) Group B: 39/49 (79.6%)	Group A:* E. coli, Enterococcus *spp.,* CNS, Klebsiella spp., S. aureus, Proteus *spp. Group B: *E. coli, Enterococcus *spp.,* S. aureus, CNS, Klebsiella *spp.	Laparotomy	No Follow-up for clinical outcomes Pre- vs. post-mobilization increase BT rates: 54/472 (11.4%), vs 39/49 (79.6%), p < 0.001	[68], **2006**
CRC (n = 158) Group A: curative surgery (n = 128) Group B: palliative surgery (n = 30)	Preoperative antibiotic treatment, unfeasible tissue sampling	MLN location: terminal ileum MLN timepoint: pre-mobilization	Culture, conventional microbial identification (counts, appearance)	Antibiotics (not specified) post sample-collection	Group A: 19/128 (15%) Group B: 4/30 (13%) Group A + B: 23/158 (14.6%)	*E. coli, Bacteroides *spp.,* E. faecalis*	Colorectal resection	Higher infectious complications BT vs. Non-BT: 9/23 (39.1%) vs. 30/135 (22.2%), p = 0.082, ns BT significantly associated with worse disease-specific and disease-free survival	[69], **2007**
Biliary malignancies (n = 51)	Intraoperative contamination (positive fat tissue)	MLN location: jejunum MLN timepoint 1: pre-mobilization 2: post-mobilization; adjacent fat tissue	RT-qPCR with primers specific to species or groups of bacteria	IV antibiotics (not specified) 30 min before surgery	Timepoint 1: 15/51 (29.4%) 2: 19/51 (37.3%)	Timepoint 1*: C. coccoides group, B. fragilis group, Enterococcus genus,* 2: *C. coccoides group, B. fragilis group, Staphylococcus genus, Enterococcus genus*	Major hepatectomy	Higher infectious complications BT vs. Non-BT post-mobilization: 11/19 (58%) vs. 5/32 (16%), p = 0.002 Lower wound infection rates BT vs. Non-BT pre-mobilization: 0/15 (0%), vs. 7/36 (19%), p = 0.07, ns Higher wound infection rates BT vs. Non-BT post mobilization: 4/19 (21%) vs. 3/32 (9%), p = 0.22, ns Higher intra-abdominal abscess rates BT vs. Non-BT pre- and post-mobilization: pre 6/15 (40%) vs. 4/36 (11%), p = 0.027, post 7/19 (37%), vs. 3/32 (9%), p = 0.023	[70], **2010**
Abdominal trauma (n = 36)	Preoperative antibiotic treatment (2 weeks), unfeasible tissue sampling, death	MLN location: terminal ileum MLN timepoint: end of surgery; serosal scraping	Culture, conventional microbial identification (counts, appearance), MLST	Antibiotics not reported	12/36 (33.3%)	*E. coli, S. epidermidis, S. aureus*	Laparotomy	Higher infectious complications BT vs. Non-BT: 5/12, (41.6%), vs. 3/24 (12.5%), p = 0.047 Higher Wound infection rates BT vs. Non-BT: 5/12 (41.6%) vs. 2/24 (8.3%), p-value not specified Higher Intra-abdominal infection rates BT vs. Non-BT: 3/12 (25%) vs. 1/24 (4.16%) p-value not specified Matching strain in MLNs and postoperative SSI (*E. coli, K. pneumoniae*) (identical five housekeeping genes)	[71], **2011**
Pancreatoduodenectomy (n = 30) Group A: standard treatment (ST) + selective decontamination digestive tract (SDD) (n = 10) Group B: ST + SDD (n = 10) Group C: ST (n = 10)	Preoperative antibiotic treatment (1 week), preoperative prebiotics (4 weeks), bilirubin > 50 mg/L	MLN location 1: proximal jejunum 2: Proximal jejunum + distal ileum MLN timepoint 1: pre-mobilization 2: post-mobilization; biopsy from jejunum, urine, blood	qPCR 16S gene, polyethylene glycol (PEG; intestinal permeability test), intestinal fatty acid-binding protein (I-FABP; mucosal damage)	IV antibiotics (Cefotaxime) 30 min before surgery SDD group: topical oropharynx + orally (Polymyxin, Tobramycin, Amphotericin B) for 4 days before surgery + IV antibiotics (Cefotaxime) for 1 day before surgery until 2 days after surgery	Timepoint 1: 13/23 (56.5%) 2: 18/27 (66.7%)	Not mentioned	Pancreatoduodenectomy	No investigations about differences in postoperative infectious complications BT vs. Non-BT No significant difference in intestinal barrier function parameters BT vs. Non-BT	[72], **2011**
CRC (n = 72) Group A: open resection (n = 35) Group B: laparoscopic resection (n = 37)	Preoperative antibiotic treatment (2 weeks), intraperitoneal sepsis/contamination, immune dysfunction, cardiac/pulmonary insufficiency	MLN location: not specified MLN timepoint 1: pre-mobilization 2: post-mobilization; liver, spleen, venous blood, urine	Culture, microbial identification (counts, appearance), bacterial endotoxin assay, lactulose/mannitol absorption test (intestinal permeability)	Antibiotics not reported	Timepoint 1: Group A: 4/35 (11.4%) Group B: 4/37 (10.8%) 2: Group A: 27/35 (77.1%) Group B: 28/37 (75.6%)	Gram-negative bacteria more common than Gram-positive bacteria, *E. coli*	Colectomy	No investigations about differences in postoperative infectious complications BT vs. Non-BT Increase of BT after bowel mobilization Group A vs. B, p < 0.05 No significant differences in BT rates Group A vs. B Increase in systemic endotoxin concentration Group A and B, p < 0.05	[73] **2013**
Esophageal cancer (n = 18)	Unfeasible tissue sampling, age over 80 years, needed 2-stage procedure	MLN location: jejunum MLN timepoint 1: pre-mobilization 2:post-mobilization; venous blood, sputum, mesenteric fat	Culture, 16S/23S RT-qPCR, sanger sequencing, sequence alignment, homology analysis from isolates, RT-qPCR with primers specific to species or groups of bacteria	IV antibiotics (Sulbactam/Ampicillin) 30 min before surgery intestinal preparation with 2L isosmotic solution 1 day before surgery	Timepoint 1: 3/18 (17%) 2: 10/18 (56%)	Timepoint 1: *Enterobacteriaceae, Atopobium cluster, B. fragilis group, C. coccoides group* Timepoint 2: *Enterobacteriaceae, Enterococcus, C. leptum subgroup, B. fragilis group, C. coccoides, Atopobium cluster*	Esophagectomy	Higher infectious complications BT vs. Non-BT: 6/10 (60%) vs. 1/8 (13%), p = 0.040 Higher SSIs BT vs. Non-BT: 3/10 (30%) vs. 1/8 (13%), p = 0.375, ns Longer hospital stays in patients with BT vs. Non-BT: 39 days ± 25 vs. 21 days ± 5, p = 0.037 High rate of 100% sequence homology *Enterobacteriaceae* MLN timepoint 2 and blood	(77), **2014**
Esophageal cancer (n = 42) Group A: synbiotics (n = 21) Group B: controls (n = 21)	Unfeasible tissue sampling, age over 80 years, needed 2-stage procedure	MLN location: jejunum MLN timepoint 1: pre-mobilization 2: post-mobilization; feces, venous blood	RT-qPCR with primers specific to species or groups of bacteria	Antibiotics not reported Synbiotics: 4 × 10^10^ living *Lactobacillus casei* strain Shirota + 1 × 10^10^ living *Bifidobacterium breve* strain Yakult + 15 g galacto-oligosaccharides daily for 7 days before surgery	Timepoint 1: Group A: 2/18 (11.1%) Group B: 4/19 (21.1%) 2: Group A: 3/18 (16.7%) Group B: 10/18 (55.6%)	Timepoint 1 Group A + B: *Enterobacteriaceae, C. coccoides group, C. leptum subgroup,* Timepoint 2 Group A: *Enterobacteriaceae, C. leptum subgroup Atopobium cluster* Group B: *Enterobacteriaceae, Enterococcus, C. leptum subgroup, B. fragilis group, C. coccoides group, Atopobium cluster, Prevotella*	Esophagectomy	No investigations about differences in postoperative infectious complications BT vs. Non-BT No significant difference in post-op infectious complications Group A vs. B Wound infection rates in Group A vs. B: 2/21 (9.52%) vs. 1/21(4.76%), p = 0.549, ns Higher BT rates in MLNs post mobilization Group B vs. A, p = 0.035 Correlation between bacteria in MLNs post-mobilization and positive bacteria in blood on post-op day 1, p < 0.001	[75], **2014**
Pancreatoduodenectomy (n = 45) Group A: synbiotics (n = 22) Group B: controls (n = 22)	Preoperative antibiotic treatment (1 week), unfeasible tissue sampling, infectious diseases	MLN location: jejunum MLN timepoint 1: pre-mobilization 2: post-mobilization; venous blood	RT-qPCR with primers specific to species or groups of bacteria	IV antibiotics (not specified) 30 min before surgery and for 3 days after surgery Synbiotics: 4 × 10^10^ living *Lactobacillus casei* strain Shirota + 1 × 10^10^ living *Bifidobacterium breve* strain Yakult + 15 g galacto-oligosaccharides daily for 7 days before surgery	Timepoint 1: Group A: 2/22 (9%) Group B: 3/22 (13.6%) 2: Group A: 4/22 (18.8%) Group B: 7/22 (31.1%)	Timepoint 1 Group A: *Enterococcus, Atopobium* Group B: *Enterobacteriaceae, Atopobium, C. coccoides* Timepoint 2 Group A*: Enterobacteriaceae, Bacteroides, C. leptum* Group B: *Pseudomonas, Streptococcus, Enterobacteriaceae, Enterococcus, Atopobium*	Pancreatoduodenectomy	No significant difference in infectious complications BT vs. Non-BT Higher infectious complications Group A vs. B: 9/22 (41%) vs. 8/22 (36%), p = 0.757, ns Higher Wound infection rates Group A vs. B: 3/22(14%), 2/22(9%), p = 0.635, ns	[76], **2016**
CRC (n = 119) Group A: open resection (n = 59) Group B: laparoscopic resection (n = 60)	Preoperative antibiotic treatment (2 weeks), intraperitoneal sepsis/contamination, immune dysfunction, cardiac/pulmonary insufficiency	MLN location: not specified MLN timepoint 1: pre-mobilization 2: post-mobilization; liver, spleen	Culture conventional microbial identification (counts, appearance), grouping latex agglutination, biochemical identification	Antibiotics not reported	Timepoint 1: Group A: 7/59 (11.8%) Group B: 7/60 (11.6%) 2: Group A: 47/59 (79.6%) Group B: 46/60 (76.6%)	Gram-negative bacteria more common than Gram-positive bacteria, *E. coli*	Colectomy	No investigation about differences in infectious postoperative complications BT vs. Non-BT Increase of BT after bowel mobilization Group A and B, p < 0.05	[77], **2015**
Esophageal cancer with neoadjuvant chemotherapy (n = 42) Group A: synbiotics (n = 20) Group B: controls (n = 22)	Preoperative use of pro- or prebiotics	MLN location: jejunum MLN timepoint 1: pre-mobilization 2: post-mobilization; feces, blood	16S/23S RT-qPCR, sanger sequencing, sequence alignment, homology analysis from isolates, YIF-Scan, RT-qPCR with primers specific to species or groups of bacteria	Antibiotics not reported Synbiotics: 4 × 10^10^ living *Lactobacillus casei* strain Shirota + 1 × 10^10^ living *Bifidobacterium breve* strain Yakult + 15 g galacto-oligosaccharides daily for 8 weeks before surgery	Timepoint 1: Group A: none Group B: 5/17 (29%) 2: Group A: none Group B: 7/17 (41%)	Group A: none Group B:* Enterobacteriaceae, Enterococcus, Streptococcus*	Esophagectomy	No investigation about differences in infectious postoperative complications BT vs. Non-BT No significant difference in the occurrence of infectious complications Group A vs. B, p = 0.608, ns Lower BT rates Group A vs. B: A: 0/38, (0%) vs. 12/34 (35.3%), p < 0.001 High sequence homology of species in MLN timepoint 2 and blood prior surgery (*E. coli, E. faecium, E. cloacae*)	[78], **2021**

^a^Rates of overall infectious complications in BT vs. Non-BT, rates of SSIs (wound infection, intra-abdominal infection, anastomotic leakage) in BT vs. Non-BT

### Characteristics and prevalence of bacteria translocating to mesenteric lymph nodes

Research on BT in late 1970s focused specifically on *E. coli*, demonstrating that high concentrations of this bacterium in the gastrointestinal tract facilitated its translocation to the MLNs [33].

Subsequent studies most frequently detected *E. coli*,* Enterococcus *spp., and *Staphylococcus *spp., followed by *Bacteroides *spp. and *Klebsiella *spp. [62–71, 73–78] (Table 1). Interestingly, these dominant bacteria in MLNs *E. coli*, *Enterococcus* spp., and *Staphylococcus* spp. are also the primary pathogens found in SSIs after abdominal surgery [31]. These facultative anaerobes are well-adapted to oxygen-rich environments, such as infected tissues with increased blood flow and hemoglobin availability [80]. Furthermore, they possess the ability to survive within professional phagocytic cells like neutrophils and macrophages [35–38, 43–49] facilitating their translocation from the intestine into systemic sites, including MLNs (Fig. 2)*.*

When interpreting the bacterial composition reported from MLNs, it's important to note that most studies relied on targeted polymerase chain reaction (PCR) with specific primers, which do not capture the full microbial diversity [70, 74–76, 78], or on conventional culture methods, which often underestimate microbial diversity and are prone to bias [62–69, 71] (Table 1). These limitations highlight the need for advanced sequencing methods to better characterize MLN-associated bacteria.

### The power of pre- and probiotics on bacterial translocation: A promising adjunct for surgical care?

The use of probiotics and synbiotics (a combination of pre- and probiotics) is a growing area of interest in perioperative care [81]. Research shows a beneficial effect of pre-and probiotics on surgical outcomes by lowering the rates of infection [82–84]. Interestingly, also the rate of BT to MLNs during surgery is reduced in patients receiving synbiotics prior to surgery [75, 76, 78]. This was assessed in three studies including patients undergoing pancreatoduodenectomy or esophagectomy (Table 1) [75, 76, 78]. These patients received a daily mix of *Lactobacillus casei*,* Bifidobacterium breve* and galacto-oligosaccharides for either 8 weeks or 7 days prior surgery [75, 76, 78]. All three studies reported a lower rate of BT in the synbiotics group [75, 76, 78]. However, none of these studies found a significant correlation between the intake of synbiotics and the reduction of postoperative infectious complications, though this was not their primary endpoint [75, 76, 78].

When evaluating why synbiotics may be beneficial in surgical patients, it is important to consider the challenges they face due to preoperative treatments. Patients with abdominal cancer undergo intensive pretreatments, including neoadjuvant oncological treatments, antibiotic therapy, and interventions.

These factors may disrupt the commensal microbiota, reduce colonization resistance, as seen in hematopoietic stem cell transplant patients [85], where risk of subsequent bacteremia is increased [85]. The loss of colonization resistance weakens both microbiota-mediated pathogen inhibition through mechanisms such as competitive exclusion and antimicrobial products and impairs innate and adaptive immune function thereby enhancing susceptibility to infections, as reviewed by Buffie et al. [86]. In healthy adults, the ability of synbiotics to successfully colonize the gut is influenced by individual factors, such as the host’s existing microbiota and immune status, with permissive individuals exhibiting lower baseline levels of probiotic strains [87]. Surgical cancer patients may be more permissive to synbiotics, as their altered microbiota could create a more receptive environment. Probiotics have the potential to restore colonization resistance [88, 89], thereby lowering the risk of BT and infection.

### Potential impact of preoperative prophylactic antibiotics on bacterial translocation

Today, the use of preoperative prophylactic antibiotics is a common practice and effectively reduces SSIs [90]. Nevertheless, it is well established that preoperative antibiotic prophylaxis, has a detrimental effect on the composition of the intestinal microbiota [91, 92]. The existing microbial composition prior to antibiotic treatment can influence how antibiotics affect microbial communities during the recovery phase, potentially leading to dysbiosis and varied recovery of the resident microbiota [91, 93]. The alteration of the residual microbial composition through antibiotic use can facilitate BT [41, 61]. The majority of human studies summarized did not specify the antibiotic agent or when it was administered (Table 1). The available evidence indicates that the incidence of SSIs and the bacterial composition observed in SSIs are significantly influenced by the preoperative administered antibiotic agent [94, 95]. We expect a similar influence of the preoperative antibiotics on the BT rate and the bacterial composition in the MLNs. However, it is important that future studies provide detailed information on the antibiotic agent and the exact schedule of administration for correct evaluation.

### Consequences of bacterial translocation to mesenteric lymph nodes: heightened risk for infectious complications

Several studies identified a significant association between the detection of bacteria in MLNs and postoperative infectious complications [63, 64, 66, 70, 71, 74, 79]. Patients with BT to MLNs exhibited 2–4.5-fold elevated rates of infectious complications (Table 1). Such infections included SSIs (superficial and deep wound infection, intra-abdominal infection and anastomosis insufficiency), bacteremia, respiratory or chest infection, and in some studies also urinary tract infection [63, 64, 66, 70, 71, 74, 79]. This association has been observed in various surgical procedures, including simple laparotomies, esophagectomies, major hepatectomies, and aortic aneurysm repairs [63, 64, 66, 70, 71, 74, 79]. Several studies have found an association between BT to MLNs and higher rates of wound infections [70, 71, 74, 79]. However, limiting factors of the studies were small sample sizes with 11 of the 18 studies including fewer than 75 patients [62, 66, 67, 70–77, 79] and overlap in patient populations in the larger studies [63–65, 68, 79].

## Factors promoting bacterial translocation during surgery

**Fig. 3:**
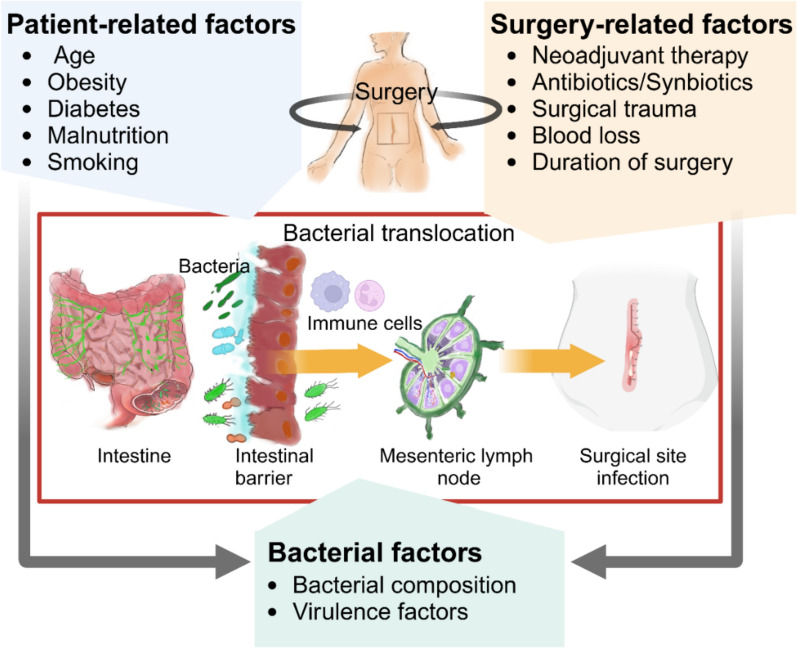
Factors influencing the risk of BT during surgery. BT is influenced by bacterial factors, such as intestinal bacterial composition and bacterial virulence factors, which affect the intestinal barrier as well as immune function. Established risk factors for SSIs, including patient-related factors such as age, obesity, diabetes, malnutrition, and smoking, as well as surgery-related factors such as neoadjuvant oncological treatments, antibiotics, synbiotics, surgical trauma, blood loss, and duration of surgery, promote BT by impacting the intestinal bacterial composition, intestinal barrier, and immune function. Created in BioRender. Zwicky, S. (2025) https://BioRender.com/egk3ku7

During surgery, specific factors promote BT and SSIs. The risk profile for BT and infection is shaped by microbial factors, patient-related comorbidities, and surgery-specific factors, all interacting in a complex manner (Fig. 3).

### Microbial factors promoting bacterial translocation during surgery

The intestinal microbial composition maintains the integrity of the intestinal barrier and limits the translocation of bacteria to the systemic circulation [41] thereby preventing infections [96–98].

In addition to the pretreatment-induced disruption of the microbiota, surgery itself induces rapid and significant changes in the intestinal bacterial composition, characterized by reduced microbial diversity and an overgrowth of Enterobacteriaceae, particularly *E. coli* and *Klebsiella *spp., by the end of the procedure [99]. These findings build upon earlier studies documenting shifts in intestinal microbiota after gastrointestinal surgery, with a later postoperative trend toward increased facultative anaerobes, as reviewed previously [100, 101]. Such rapid changes in the intestinal composition during surgery could be influenced by a variety of factors including previously disturbed microbiota, the preoperative fasting period [102] and the preoperative single-shot antibiotic prophylaxis, both of which decrease the microbial biomass and the overall microbial diversity [91, 92, 102]. Mechanical ventilation during surgery results in hyperoxygenation, potentially decreasing the population of anaerobic bacteria while enhancing the growth of aerobic and facultative anaerobic bacteria, such as the especially oxygen-tolerant Enterobacteriaceae family [103, 104]. Such disruptions in intestinal microbiota by the end of surgerymay facilitate BT [41].

Bacterial virulence factors are crucial for bacterial survival in MLNs and the establishment of infection [39, 40, 55–57]. The evidence regarding whether surgery itself leads to the acquisition of virulence factors is limited. One study suggests that phosphate depletion in the intestinal mucosa during surgery  leads *P. aeruginosa* to upregulate virulence factors, such as PstS, a phosphate-scavenging protein, leading to increased sepsis rates and mortality in a partial hepatectomy model [105]. Further research is needed to better understand how virulence factors are acquired during surgery.

### Patient- and surgical-related factors promoting bacterial translocation

Patient- as well as surgical-related risk factors for SSIs [11–14, 106] may contribute to BT during surgery by influencing microbial composition, intestinal integrity, and immune function. The most common patient-related risk factors for SSIs include advanced age, obesity, diabetes, malnutrition, and cigarette smoking [11–14, 106]. Older age is linked to microbial dysbiosis, which contributes to increased intestinal permeability and BT as well as to heightened inflammation and impaired macrophage function [107]. Obesity is associated with barrier dysfunction and translocation [108]. Diabetes and hyperglycemia can also disrupt the intestinal epithelial barrier [109]. Lifestyle factors such as diet [110, 111] and exercise [112] play a crucial role in shaping the intestinal microbiota. In particular, low fiber [113] and malnutrition compromise the intestinal barrier [114] and induce intestinal dysbiosis [110]. Yet, more than a third of patients undergoing elective abdominal cancer surgery are severely malnourished, significantly increasing the risk of SSIs [106]. Cigarette smoking increases the risk of SSIs [115] through various mechanisms, including a compromised intestinal barrier [116] and intestinal dysbiosis [117, 118]. Surgical trauma-induced immune suppression [119–122], worsened by prolonged surgery and significant blood loss, increases the risk of BT and infection. Significant blood loss during surgery can additionally cause splanchnic hypoperfusion, resulting in intestinal injury and increased permeability [123].

In conclusion, BT during surgery results from a complex interplay of microbial, patient-related, and surgical factors. While surgical factors are difficult to modify, optimizing patient-related factors, such as preoperative weight management, exercise, glycemic control, nutrition, and smoking cessation could reduce BT and infections [115, 124].

## Technical limitations in bacterial translocation research and future perspectives

**Fig. 4:**
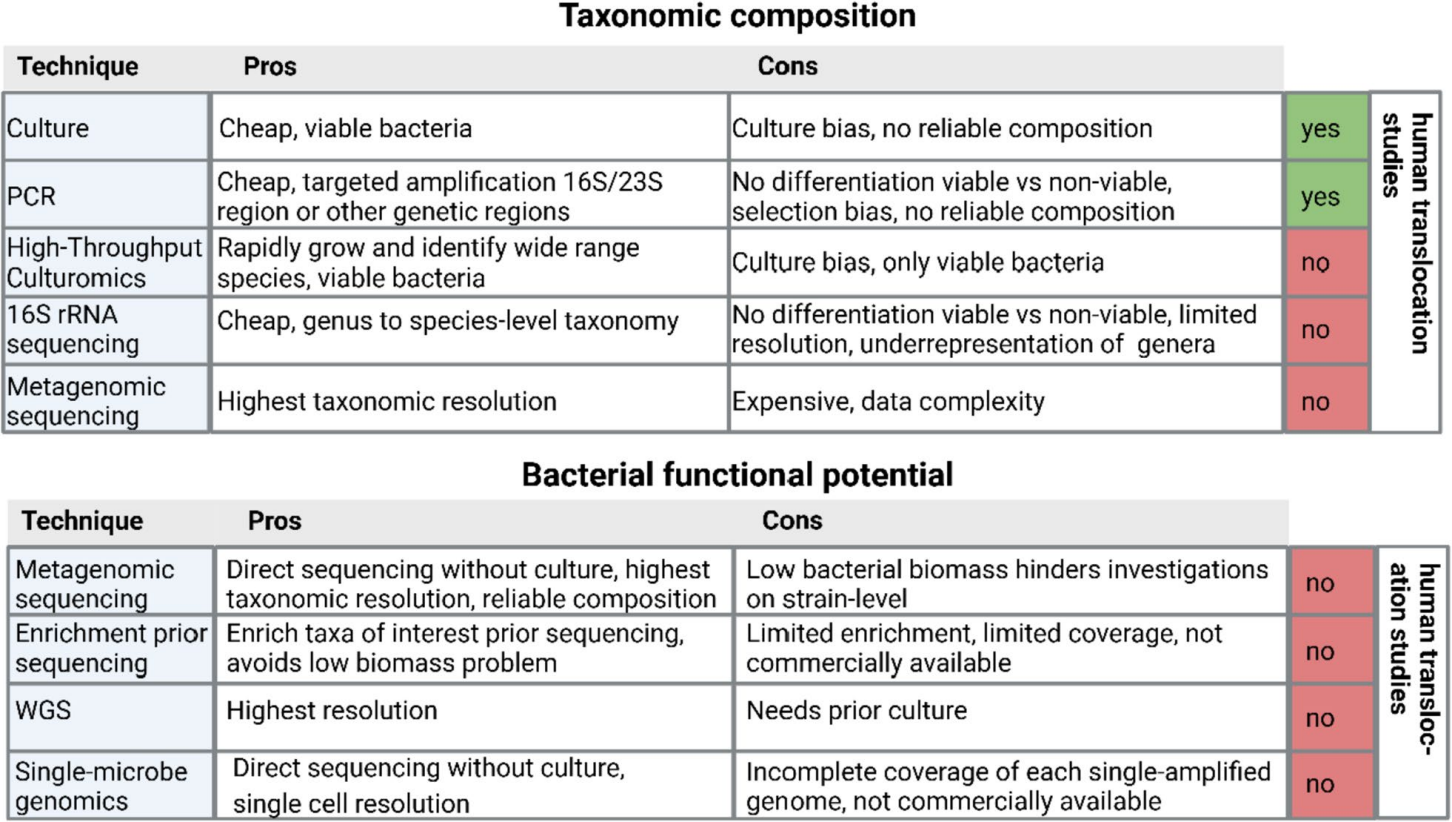
Current and emerging techniques for studying BT to MLNs. Comparison of the advantages and limitations of current methods used in human studies to investigate BT to MLNs. The figure also highlights novel techniques with the potential to enhance the exploration of bacterial composition and the bacterial genetic potential at the intestine, MLNs, and infected sites. Created in BioRender. Zwicky, S. (2025) https://BioRender.com/xvrx8wr

BT to MLNs has been known for nearly 50 years [33] and associations to infectious complications have been suggested since the end of the twentieth century. Nevertheless, research in this field has largely stagnated over the past decades, partly due to the inherent limitations of available techniques (Table 1) [62–79]. This section examines the advantages and disadvantages of the techniques used in existing studies (Table 1), and explores how novel, cutting-edge methods could drive future research advancements (Fig. 4).

### Techniques employed in human studies investigating bacterial translocation to mesenteric lymph nodes to date

Many studies have relied on culture-based methods to detect viable bacteria [62–69, 71, 73, 74, 77, 79] (Table 1). Culturing methods offer the advantage of identifying viable bacteria and the isolates could be used in further experiments [125]. Despite their utility, culture-based methods have notable limitations, including low sensitivity and selective biases that underestimate BT incidence and microbial diversity. Many fastidious or anaerobic species fail to grow under standard conditions and dominant taxa may overshadow low-abundance or slow-growing organisms [125]. This may lead to a distorted representation of the actual microbiota involved in BT. Additionally, these methods are labor-intensive and time-consuming thus limiting their scalability [125].

Building on the limitations of culture-based methods, PCR has emerged as a more sensitive alternative for detecting bacterial DNA or RNA in MLNs, addressing some of the gaps in microbial detection, such as the identification of non-culturable or slow-growing bacteria that may be missed by traditional culture techniques. [67, 70, 72, 74–76, 78]. Studies used quantitative PCR (qPCR) to quantify bacterial DNA, specifically the 16S ribosomal RNA (rRNA) gene [67, 72], or employed reverse transcription-qPCR (RT-qPCR) to target bacterial rRNA transcripts [70, 74–76, 78]. These methods either quantified the general presence of bacterial DNA [72] or utilized specific primers to detect bacterial species or groups, such as *Staphylococcus*, *Enterococcus*, or *Enterobacteriaceae* [67, 70, 74–76, 78].

While these methods have the ability to identify particular taxa, they are limited in their scope to capture microbial diversity [67, 70, 74–76, 78].

Two studies used RT-qPCR to amplify 16S and 23S rRNA gene fragments and then sequenced the fragments to compare bacteria found in MLNs and blood-samples of the same patient [74, 78]. While this method gives additional insights in genetic similarity, information on the stain level is lost as only the 16S and 23S rRNA genes are analyzed [74, 78].

Multilocus sequencing typing (MLST) was employed to analyze five housekeeping genes from isolates obtained from different sites within the same patient in one study [71]. While this approach is the closest among the studies for determining whether similar strains are present in the MLNs and infected sites, MLST still lacks the resolution needed to distinguish between many bacterial strains within a species [126]. Advanced techniques such as metagenomic sequencing and whole genome sequencing (WGS) could overcome such challenges by enabling the comparison of complete genomes as discussed below [126–129].

### Advancing the identification of bacterial composition in clinical samples

Although culture and RT-qPCR are effective for identifying bacteria, they are limited in assessing the full taxonomic composition of a clinical sample. Since the 2000s, 16S rRNA gene sequencing has been the core technology for bacterial identification and classification [130, 131]. Although 16S rRNA sequencing provides valuable insights into the presence and abundance of bacterial taxa, it generally offers lower taxonomic resolution, and can underrepresent several low-abundance phyla compared to metagenomic sequencing [132]. Additionally, the choice of the primers determines which region of the 16S-gene (V1–V9) is amplified. This affects the taxonomic composition and can introduce a bias by preferentially amplifying certain bacteria over others [133]. To achieve a more detailed, and reliable, taxonomic resolution, down to the species or even strain level from a complex clinical sample, metagenomic sequencing could be applied [127, 134, 135]. Advanced bioinformatics tools and improved sequencing technologies further strengthen its utility, offering higher accuracy and resolution [136], which are essential for tackling complex microbiological questions in clinical and research settings.

### Identification of the bacterial functional potential with cutting-edge techniques

Metagenomic sequencing also provides information on the functional potential of the microbiota. Since the entire genome is sequenced, it is possible to focus on several genes of interest for example those conferring antibiotic resistance or virulence factors [127–129]. However, metagenomic sequencing of clinical samples faces several challenges. Besides the relatively high costs, a major limitation is the contamination of bacterial DNA with host DNA, which can hinder a strain or sub-strain level analysis [127]. Although a reliable taxonomic composition might still be possible, high levels of host DNA hinder the de novo assembly of the metagenome, making a thorough functional genetic analysis challenging or even impossible [127]. Therefore, efficient separation of microbial DNA from host DNA is necessary to improve the accuracy and reliability of metagenomic studies [127]. The use of host-depletion kits to extract the DNA from low biomass microbial samples like MLNs can enrich the proportion of bacterial DNA by up to 30% [127, 137, 138]. A study reported even a reduction of the 18S/16S rRNA ratio by 32- and 57-fold when comparing different host depletion kits [138]. However, since samples from human tissues such as MLNs typically contain less than 1% of bacterial DNA, this enrichment alone is insufficient to investigate functional properties, regardless of the metagenomic sequencing depth [127]. WGS provides an effective alternative for studying genetic functions in single isolates from culture, offering the highest resolution without interference from host DNA [128].

### Bacterial enrichment to reduce host-associated noise

Alternative methods to enrich specific bacterial genomic regions [27] or bacterial taxa of interest [139] and thereby deplete host and background taxa are emerging: a recent study aimed to determine if pathogens found in SSIs after spine surgery are already present in the preoperative microbiome [27]. The study used isolate-specific probes from pathogens found in SSIs to selectively enrich targeted bacterial DNA from preoperatively collected oral, nasal and rectal swabs [27]. While this approach could be also valuable for studying the role of BT in SSI development in the future, this study has several limitations. Not all bacteria of interest could be enriched, and the extent of enrichment was comparable to that achieved with host depletion kits. When comparing the similarity of SSI pathogens with preoperative metagenomic reads, the resolution of the metagenomic sequencing was too low to build metagenome-assembled genomes, so a reference-based strategy was used. Even a single metagenomic read matching an SSI-pathogen specific strain-informative polymorphism (SIP) was considered as coverage. From the covered SIPs, allelic concordance was calculated and reported as the fraction of all covered SIPs. Concordance of > 80% was considered indicative of genomic similarity to the preoperative strain, classifying the bacteria as endogenous. However, the number of covered SIPs varied widely across different patients, from 1 to 24,731. Cases with limited site coverage (e.g., only 10 sites) and > 80% concordance were still classified as endogenous, raising concerns about the reliability of such conclusions [27].

Relying on only a single read to cover a SIP introduces uncertainty. Single-read coverage is susceptible to sequencing variability or random noise, making it difficult to confidently report endogenous strain identity, particularly when only a limited number of SIPs are analyzed. Despite these limitations, the study by Long and colleagues is currently one of the most advanced investigations regarding the potential endogenous origins of bacteria found in SSIs [27]. Furthermore, the study highlights that optimization of enrichment techniques is urgently needed, which could significantly enhance future investigations into BT and its role in SSI pathogenesis.

### Illuminating the origin and function of pathogens causing surgical infections by combining techniques

A promising approach for achieving both high-resolution taxonomic identification and comprehensive genetic insights into pathogens associated with SSIs is the combination of metagenomic sequencing [127] and WGS of cultured isolates [134]. WGS of single isolates allows a comprehensive analysis of the entire genome with high coverage, facilitating the investigation of specific genes, mutations, and genomic features linked to pathogenicity and antibiotic resistance [134].

A critical first step will be the establishment of a reliable, species-level characterization of microbial communities across key anatomical sites during surgery, including the systemic circulation and SSIs, as this remains a significant gap in current research. To pinpoint the origin of individual bacterial strains responsible for SSIs, comprehensive strain-level analyses should be performed, comparing pathogens isolated from SSIs with those from other patient sites. Whole-genome analysis could further enhance this understanding by identifying specific genetic loci linked to BT and infection, offering valuable insights into the mechanisms driving these processes [128, 129, 134].

More recent methods advance such a combination of techniques: high-throughput microbial culturomics facilitates culturing by combining automation and machine learning to rapidly culture and identify a wide range of bacterial species, including difficult-to-culture ones [140]. Another novel method is single-microbe genomics, where individual microbes are captured in liquid droplets and barcoded for whole-genome amplification. However, a current limitation of this technique is the incomplete coverage of each single-amplified genome [141]. Although combining genetic information from multiple microbes of the same strain can enhance coverage, this approach may mask critical differences between sub-strains [141].

In the future, a combination of these advanced techniques holds the promise to provide precise information about the origin of pathogens involved in the development of SSIs and their route of infection.

## Conclusion

SSIs remain a significant challenge in postoperative care, particularly following abdominal surgeries [6–9]. Recent evidence underscores the significant role of the patient’s own microbiota in the pathogenesis of SSIs [27]. BT from the intestine to normally sterile secondary sites has emerged as a pivotal mechanism contributing to infections [63, 64, 66, 70, 71, 74, 79]. This process occurs through both intracellular pathways and extracellular routes, highlighting the complexity of bacterial dissemination during surgery.

Although over 20 years of research have established the association between BT to MLNs and increased risks of infectious complications [63, 64, 66, 70, 71, 74, 79], progress in this field has been slow. Limitations in methodologies, including reliance on culture-based techniques and PCR approaches, have hindered comprehensive understanding. Advanced sequencing technologies, such as metagenomics and WGS, offer promising avenues to investigate the microbial composition and genetic characteristics of bacteria in MLNs and SSIs. However, challenges such as host DNA contamination and insufficient resolution at the strain level persist, necessitating further optimization of these techniques.

By leveraging cutting-edge methodologies and focusing on patient-specific microbial profiles, this research has the potential to uncover novel targets for personalized preventive and therapeutic strategies, ultimately reducing the burden of SSIs and improving surgical outcomes.

## Data Availability

Not applicable.

## Abbreviations

SSIs: Surgical site infections
BT: Bacterial translocation
MLNs: Mesenteric lymph nodes
*E. coli*: *Escherichia coli*
*S. aureus*: *Staphylococcus aureus*
*E. faecalis*: *Enterococcus faecalis*
*K. pneumoniae*: *Klebsiella pneumoniae*
*K. oxytoca*: *Klebsiella oxytoca*
*C. coccoides*: *Clostridium coccoides*
*C. leptum*: *Clostridium leptum*
PS: Phosphatidylserine
MRSA: Methicillin-resistant *S. aureus*
*S. typhimurium*: *Salmonella typhimurium*
*S. pyogenes*: *Streptococcus pyogenes*
*P. aeruginosa*: *Pseudomonas aeruginosa*
SpyCEP: *S. pyogenes* Cell-Envelope Proteinase
*C. perfringens*: *Clostridium perfringens*
*B. fragilis*: *Bacteroides fragilis*
PCR: Polymerase chain reaction
qPCR: Quantitative PCR
rRNA: Ribosomal RNA
RT-qPCR: Reverse transcription -qPCR
MLST: Multilocus sequencing typing
WGS: Whole genome sequencing
SIP: Strain-informative polymorphism
CRC: Colorectal cancer
CNS: Coagulase-negative staphylococci
*E. cloacae*: *Enterobacter cloacae*
*M. morganii*: *Morganella morganii*
*S. epidermidis*: *Staphylococcus epidermidis*
*P. mirabilis*: *Proteus mirabilis*
*S. mitis*: *Streptococcus mitis*
ST: Standard treatment
IV: Intravenous
SDD: Selective decontamination of the digestive tract
ST: Standard treatment
PEG: Polyethylene glycol
I-FABP: Intestinal fatty acid-binding protein
